# NADPH Oxidase Isoforms in COPD Patients and Acute Cigarette Smoke-Exposed Mice: Induction of Oxidative Stress and Lung Inflammation

**DOI:** 10.3390/antiox11081539

**Published:** 2022-08-08

**Authors:** Xinjing Wang, Priya Murugesan, Pan Zhang, Shiqing Xu, Liang Peng, Chen Wang, Hua Cai

**Affiliations:** 1 Department of Pulmonary and Critical Care Medicine, China-Japan Friendship Hospital, Capital Medical University, Beijing 100069, China; 2 Department of Anesthesiology, Department of Medicine, David Geffen School of Medicine, University of California Los Angeles, Los Angeles, CA 90095, USA; 3 Chinese Academy of Medical Sciences and Peking Union Medical College, Beijing 100730, China

**Keywords:** chronic obstructive pulmonary disease (COPD), NADPH oxidase (NOX), NOX1, NOX2, NOX4, NOX5, acute cigarette smoke (ACS), oxidative stress, lung inflammation

## Abstract

Cigarette smoke (CS) is a major risk factor for chronic obstructive pulmonary disease (COPD), which represents the third leading cause of death worldwide. CS induces reactive oxygen species (ROS) production, leading to pulmonary inflammation and remodeling. NADPH oxidases (NOXs) represent essential sources of ROS production in the cardiovascular system. Whether and how NOX isoforms are activated in COPD patients and in response to acute cigarette smoke (ACS) remains incompletely understood. In the present study, the expression of NOX isoforms was examined in the lungs of end-stage COPD patients. In addition, mice silenced of NOX1 or NOX4 expression using in vivo RNA interference (RNAi), and NOX2-deficient (NOX2^−/y^) mice, were exposed to ACS for 1 h using a standard TE-10B smoking machine. In lung sections isolated from COPD patients undergoing lung transplantation, protein expression of NOX1, NOX2, NOX4, or NOX5 was markedly upregulated compared to non-smoking donor controls. Likewise, ACS upregulated protein expression of NOX1, NOX2, and NOX4, production of ROS, inflammatory cell infiltration, and mRNA expression of proinflammatory cytokines TNF-α and KC in the mouse lung. In vivo RNAi knockdown of NOX1 or NOX4 decreased ACS induced ROS production, inflammatory cell influx, and the expression of TNF-α and KC, which were accompanied by inhibition of the NF-κB-COX-2 axis. Although ACS induced ROS production was reduced in the lungs of NOX2^−/y^ mice, inflammatory cell influx and expression of NF-κB/COX-2 were increased. Taken together, our results demonstrate for the first time that NOX isoforms 1, 2, 4 and 5 all remain activated in end-stage COPD patients, while NOX1 and NOX4 mediate oxidative stress and inflammatory responses in response to acute cigarette smoke. Therefore, targeting different isoforms of NOX might be necessary to treat COPD at different stages of the disease, which represents novel mechanistic insights enabling improved management of the devastating disease.

## 1. Introduction

Chronic obstructive pulmonary disease (COPD) is characterized by progressive and irreversible airflow limitation caused by chronic bronchitis and emphysema [1]. Of note, COPD is a major global health problem and the third leading cause of death worldwide. In 2017, 3.2 million deaths worldwide were attributed to COPD, and the annual death toll is expected to increase to 4.4 million by 2040 [2]. Oxidative stress and persistent inflammation are major pathogenic mechanisms of COPD [3]. In addition, oxidative stress has been shown to play an important role in driving inflammatory responses during the early stage of COPD development [4,5].

Cigarette smoke (CS) contains thousands of hazardous chemical compounds, many of which have been shown to have acute effects on oxidative and inflammatory responses in vitro and in vivo [6,7]. It is generally recognized that CS is one of the most important risk factors for the development of COPD, and it is associated with 80–90% of COPD cases in the United States [8,9]. CS is also a major stimulator of reactive oxygen species (ROS) production in patients with COPD [8]. It has been shown that CS can directly activate airway surface macrophages and epithelial cells to release ROS and multiple chemotactic mediators, which in turn attract circulating monocytes and neutrophils into the lung [5]. Nonetheless, the enzymatic sources of ROS production in the lung in response to acute cigarette smoke (ACS) exposure have remained incompletely understood.

NADPH oxidases (NOXs) are the main cellular sources of ROS and have been shown to play important roles in mediating a variety of pathological conditions, especially in the pathogenesis of cardiovascular diseases [10,11,12,13,14]. Several recent studies have revealed that each isoform of the NOX family might exist in different types of cells in the lung and is responsible for tissue damage associated with several lung diseases [15,16,17]. Activation of NOX2 and NOX4 isoforms is primarily associated with increased ROS production in asthma, which has been demonstrated in both patients and animal models [18,19]. Both of these isoforms have also been implicated in pulmonary hypertension [20,21]. NOX4-derived ROS play a vital role in epithelial cell death and fibroblast differentiation, leading to pulmonary fibrosis [22,23,24]. Interestingly, Schiffers et al. recently demonstrated downregulation of DUOX1 in COPD, indicating a protective role of DUOX1 [25,26], which is consistent with the findings of Nagai et al. [27]. On the other hand, NOX2 knockout in mice was found either to effectively attenuate ROS production while aggravating inflammatory responses and emphysema, or to protect against elastase-induced emphysema [28,29]. Of note, deletion of the NOX1 binding subunit NOXO1 in mice was recently found to be protective against emphysema [30]. Nevertheless, the potential differential roles of NOX isoforms NOX1–5 in the pathogenesis of COPD remain incompletely understood, especially in COPD patients and ACS induced oxidative stress and inflammatory responses.

Therefore, in the present study, we first examined changes in the protein expression of NOX isoforms NOX1, NOX2, NOX4, and NOX5 in lung sections of human COPD patients undergoing lung transplantation; and then investigated the roles of NOX isoforms in regulating oxidative stress and lung inflammation in mice exposed to acute cigarette smoke. Compared to the donor group, the protein expression of NOX isoforms NOX1, NOX2, NOX4, and NOX5 was significantly upregulated in lung tissue sections of patients with end-stage COPD. In mice exposed to ACS using a standard TE-10 smoking machine, the protein expression of NOX1, NOX2, and NOX4 in lung tissues was significantly upregulated. In mice silenced of NOX1 or NOX4 expression using in vivo RNAi, ACS induced ROS production, inflammatory cell infiltration, and expression of TNF-α and KC were significantly alleviated, which were accompanied by inhibition of NF-κB and COX-2. Although ACS induced ROS production was attenuated in the lungs of NOX2^−/y^ mice, inflammatory cell infiltration and expression of inflammatory cytokines were increased, which may be attributed to an intrinsic propensity for exaggerated inflammation in NOX2^−/y^ mice.

## 2. Materials and Methods

### 2.1. Collection of Human Lung Tissues of COPD Patients and Healthy Donors Undergoing Lung Transplantation

Lung tissue specimens from 7 healthy donors and 18 COPD patients undergoing lung transplantation were collected at the Center of Lung Transplantation, China-Japan Friendship Hospital, Beijing, China. All of the healthy donors were life-long non-smokers, while COPD patients were all former/current smokers. The diagnosis of COPD was confirmed according to the GOLD (Global Initiative for Chronic Obstructive Lung Disease) criteria (post-bronchodilator FEV1/FVC < 70%) [31], and the basic characteristics of healthy donors and COPD patients are shown in Table 1. The experiments were approved by the Institutional Review Board.

### 2.2. Immunohistochemistry (IHC) of Human Lung Sections

Formalin-fixed, paraffin-embedded lung sections (3 µm thick) from healthy donors and COPD patients were deparaffinized in xylene and rehydrated in a graded ethanol series. Heat-induced epitope retrieval was performed by boiling the slides in citrate buffer. After cooling at room temperature, the tissue sections were incubated with 3% hydrogen peroxide for 20 min, and then the nonspecific sites were blocked with 10% goat serum for 30 min. The slides were then incubated with antibodies for NOX1 (1:100, Novus Biologicals, Littleton, CO, USA), NOX2 (1:100, Santa Cruz Biotechnology, Santa Cruz, CA, USA), NOX4 (1:200, Novus Biologicals), or NOX5 (1:1000, Abcam, Cambridge, UK) in a humidified chamber at 4 °C overnight and washed three times with PBS. The slides were incubated with HRP-conjugated goat anti-rabbit or anti-mouse secondary antibodies, rinsed in PBS, and stained using the DAB Substrate Kit (Cell Signaling Technology, Danvers, MA, USA) with counterstaining of hematoxylin. The intensities of each NOX isoform in the lung sections were quantified using randomly selected images showing structures of alveolars, small airways, and pulmonary arteries in ten fields per subject with the Image Pro Plus 6.0 software (Media Cybernetics, Bethesda, MD, USA).

### 2.3. Animals

Eight-week-old male C57BL/6 mice were purchased from Charles River China Inc. (Beijing, China) and used as wild-type (WT) control mice. Age-matched NOX2^−/y^ mice (C57BL/6 strain background, Strain #: 002365) were purchased from Jackson Laboratories (Bar Harbor, ME, USA) and maintained in Huafukang Bioscience Co., Inc. (Beijing, China). Under specific pathogen-free conditions, all mice were maintained on a 12 h light/dark cycle with free access to food and water. Mice of the right age were transferred and used for experiments, with all procedures approved by the Research Ethics Committee of China-Japan Friendship Hospital. In parallel experiments, WT C57BL/6 mice of 8 weeks old were silenced of NOX1 or NOX4 expression using in vivo RNA interference (see below).

### 2.4. Exposure of Mice to Acute Cigarette Smoke (ACS)

The mice were exposed to ACS using a standard TE-10B smoking machine (Teague Enterprises, Davis, CA, USA) by burning Hongtashan cigarettes (1.1 mg of nicotine, 11 mg of tar, and 11 mg of carbon monoxide per cigarette; Hongta Group, Yuxi, China), as described previously [32]. Eight cigarettes were smoked at a time using the puffing method for 2 s once per minute at a volume of 35 cm^3^. Cigarettes were smoked for 8 min each. The chamber atmosphere was monitored for total suspended particulates with a particulate monitor (pDR-1500, Thermo Fisher Scientific, Waltham, MA, USA) at a steady concentration of 150 mg/m^3^. The mice were exposed to cigarette smoke (CS) or filtered air for 1 h and then sacrificed 6 hrs later for histological analyses and examination of ROS production and inflammatory responses. Upon harvest, the left lung was perfused and formalin-fixed for histologic evaluation. The upper lobe of the right lung was perfused and embedded in optimal cutting temperature (OCT) compound for fluorescence imaging analyses; the other 3 lobes of the right lung were stored at −80 °C for analyses of mRNA and protein expression.

### 2.5. In Vivo RNA Interference-Mediated Knockdown of NOX1 and NOX4

siRNA specifically designed to target mouse NOX1 or NOX4 was purchased from Dharmacon (Chicago, IL, USA) with the sequences [5′- > 3′] NOX1: GCUGGUGGCUGGUGA CGAAUU; NOX4: CAUGCUGCUGCUGUUGCAUGUUUCA). siRNAs were prepared in Invivofectamine 3.0 reagent (Invitrogen, Carlsbad, CA, USA) for in vivo delivery as we previously published [33]. In brief, 100 μL of 1.2 mg/mL siRNA solution was mixed with 100 μL Invivofectamine 3.0 reagent. After incubation at 50 °C for 30 min, the mixture was diluted six-fold by adding 1 mL of phosphate-buffered saline (PBS). Subsequently, this mixture was injected into mice via the tail vein (1 mg siRNA/kg body weight) 24 h before exposing mice to ACS. Liposome-based transfection reagents have been shown previously to successfully deliver siRNA to the mouse lung to attenuate gene expression [34].

### 2.6. Histological Analyses of Mouse Lung Tissue Sections

For histological analyses, freshly isolated lung tissues were immersed in fresh 10% formalin for at least 24 h, after which they were embedded in paraffin and sectioned (3 µm thick). The lung sections were stained with hematoxylin and eosin (H&E) following the standard protocol and examined under an optical microscope (Olympus Optical, Tokyo, Japan). These lung sections were used to assess inflammatory cell infiltration in the alveolar spaces. Ten fields per section were randomly selected for each mouse and imaged at 100× magnification.

### 2.7. Bronchoalveolar Lavage Fluid (BALF) Collection and Cell Counting

Six h after CS exposure, mice were anesthetized with intraperitoneal pentobarbital (50 mg/kg; Abbott Laboratories, Abbott Park, IL, USA). BALF was obtained by cannulating the trachea and injecting and retracting 1 mL of cold PBS three times, and it was centrifuged at 3000 rpm for 10 min at 4 °C. The pellet of BALF was resuspended in erythrocyte lysis buffer (Beyotime, Shanghai, China) for 2 min and centrifuged again. Subsequently, the pelleted cells were washed twice with 500 µL of PBS and then resuspended in 300 µL of PBS. The total cell count was obtained using a hemocytometer (Hausser Scientific, Horsham, PA, USA). The cell suspension was then placed on slides and stained with Giemsa (Solarbio, Beijing, China). The distribution of macrophages and neutrophils was determined by counting at least 300 total cells for each slide. The number of each cell type was calculated by multiplying the percentage by the total number of cells. 

### 2.8. Determination of ROS Production in Mouse Lungs

In brief, the superior lobes of right lungs freshly isolated from mice were immersed in Tissue Plus^®^ OCT compound (Sakura Finetek, Torrance, CA, USA) and then frozen at −20 °C and sectioned (5 µm thick). The superoxide production in lung sections was assessed by dihydroethidium (DHE) fluorescent imaging. The lung sections were incubated with 2 μM DHE (MilliporeSigma, St. Louis, MO, USA) for 30 min in the dark and washed three times with Krebs–Henseleit Bicarbonate buffer. The sections were then mounted with ProLong Gold Antifade (Invitrogen, Waltham, MA, USA) and imaged using a Nikon A1R confocal microscope (Nikon, Tokyo, Japan). Images were captured and analyzed using the NIH Image J software.

### 2.9. Reverse Transcription Quantitative Polymerase Chain Reaction (RT-qPCR)

Total RNA was extracted from the lung tissues of mice using TRIzol reagent (Invitrogen, Waltham, MA, USA). The RNA concentration was determined using a NanoDrop One spectrophotometer (Thermo Fisher Scientific, Waltham, MA, USA). cDNA was synthesized from 2 μg of RNA using the High-Capacity cDNA Reverse Transcription Kit (Applied Biosystems, Cheshire, UK). PCR was performed using SsoFast EvaGreen supermix (Bio-Rad, Hercules, CA, USA) with a CFX96 real-time PCR detection system (Bio-Rad). Relative quantification of mRNA expression for each gene was calculated using the 2^−ΔΔCt^ method, with mouse β-actin used as an internal reference gene. The following primers were used: tumor necrosis factor α (TNF-α) forward: TCTGTCTACTGAACTTCGGGGTGA, reverse: TTGTCTTTGAGATCCATGCCGTT; keratinocyte-derived chemokine (KC) (mouse homolog of human IL-8) forward: ACCCAAACCGAAGT CATAGCC, reverse: TTGTCAGAAGCCAGCGTTCA; β-actin forward: CACTGTGC CCATCTACGA, reverse: GTAGTCTGTCAGGTCCCG.

### 2.10. Immunohistochemistry

The fixed lung sections (3 µm thick) were deparaffinized in xylene and rehydrated in a graded ethanol series until water was used. Heat-induced epitope retrieval was performed by boiling the slides in citrate buffer. After cooling at room temperature, the tissue sections were incubated with 3% hydrogen peroxide for 20 min, and then the nonspecific sites were blocked with 10% goat serum for 30 min. Without washing, the following primary antibodies were applied for detection: NF-κB p65 (1:800, Cell Signaling Technology, Danvers, MA, USA) and phospho-NF-κB p65 (1:100, Cell Signaling Technology, Danvers, MA, USA). The slides were incubated with the primary antibodies in a humidified box at 4 °C overnight. After incubating with HRP-conjugated goat anti-rabbit secondary antibody, the sections were stained with the DAB Substrate Kit (Cell Signaling Technology, Danvers, MA, USA) and counterstained with hematoxylin. The intensities of the target proteins were quantified using the Image Pro Plus 6.0 software.

### 2.11. Western Blotting

Total proteins in the lungs were extracted using protein lysis buffer (20 mM Tris-HCL, 150 mM NaCl, 1 mM EDTA, 1 mM EGTA, 2.5 mM Sodium pyrophosphate, 1.22 mM MgSO_4_, 20 mM Tris-HCL, and 150 mM NaCl) containing protease inhibitor cocktail (MilliporeSigma, Burlington, MA, USA). Protein concentration was determined using a bicinchoninic acid assay kit (Cell Signaling Technology, Danvers, MA, USA). Aliquots of protein extract (40 μg per lane) were separated in 10% sodium dodecyl sulfate–polyacrylamide gel electrophoresis, transferred to polyvinylidene fluoride (PVDF) membranes (MilliporeSigma, Burlington, MA, USA), and then blocked with 5% bovine serum albumin in TBST (mixture of Tris-buffered saline and 0.1% Tween 20) for 1 h. Then, the membranes were incubated overnight at 4 °C with primary antibodies against p65, p-p65, p-IKKα/β, IκBα or β-actin (all at 1:1000, Cell Signaling Technology, Danvers, MA, USA), COX-2 (1:200, Cayman Chemical, Ann Arbor, MI, USA), NOX1 or NOX4 (both at 1:500, Novus Biologicals, Littleton, CO, USA), or NOX2 (1:100, Santa Cruz Biotechnology, Dallas, TX, USA), followed by incubation with anti-mouse or anti-rabbit secondary antibody for 1 h at room temperature. The bands were visualized with the enhanced chemiluminescence Plus detection reagent using the ChemiDoc XRS+ system (Bio-Rad), and quantified using the NIH Image J software.

### 2.12. Statistical Analysis

All data are presented as Mean ± SEM. Statistical analysis was performed using GraphPad Prism 7.0 (GraphPad Software, San Diego, CA, USA). Data were analyzed by Student’s *t* test (unpaired) for comparisons of two groups or by one-way ANOVA followed by Tukey’s post hoc test for comparisons of multiple groups. All experiments were repeated independently three to five times, and the numbers of repeats for each experimental group are included in the results section and figure legends. *p* < 0.05 was considered statistically significant.

## 3. Results

### 3.1. The Protein Expression of NOX Isoforms NOX1, NOX2, NOX4, and NOX5 Was Significantly Upregulated in Lung Tissue Sections of Patients with End-Stage COPD

**Figure 1:**
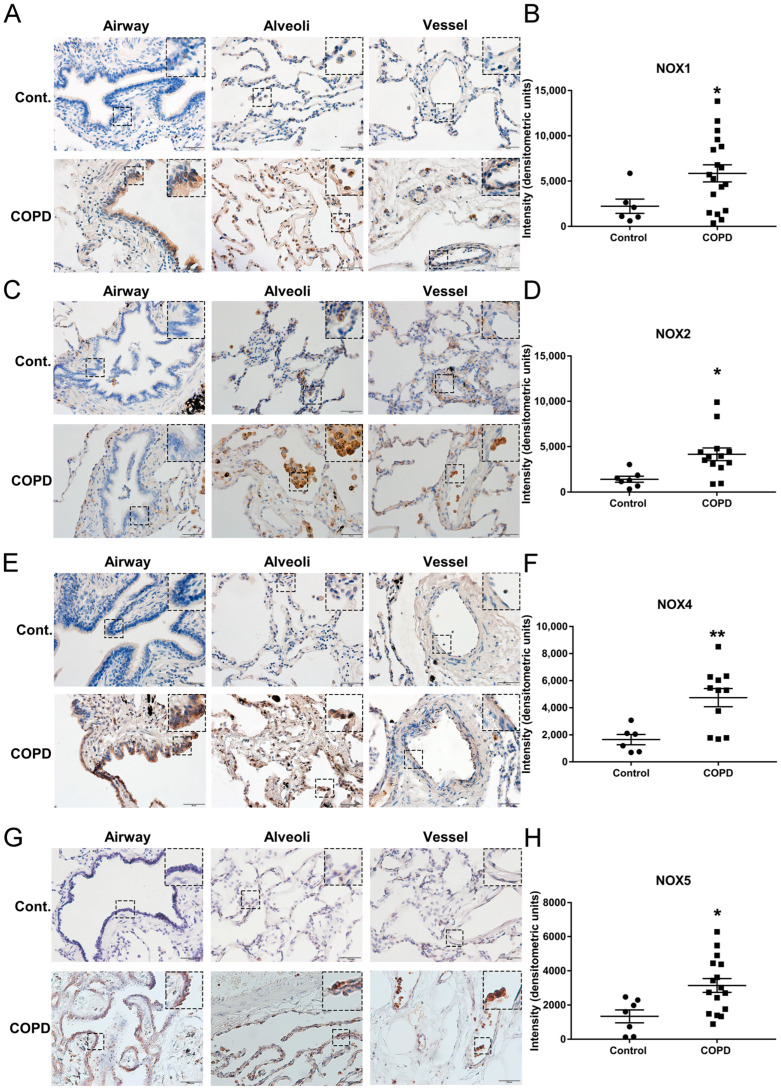
**Upregulation of NOX1, NOX2, NOX4, and NOX5 in lung tissue sections of patients with end-stage COPD**. Lung tissue sections of end-stage COPD patients and non-smoking donor controls were subjected to immunohistochemical staining using antibodies for NOX1, NOX2, NOX4, or NOX5. The representative images are shown in panels (**A**,**C**,**E**,**G**), while the grouped data are shown in panels (**B**,**D**,**F**,**H**). The data indicate that the protein expression levels of NOX1, NOX2, NOX4, and NOX5 were significantly upregulated in end-stage COPD patients compared to donor controls. All data are presented as Mean ± SEM, *n* = 6–18. Scale bar, 50 µm. * *p* < 0.05; ** *p* < 0.01 vs. donor control group.

In order to evaluate whether NOX-derived oxidative stress might be involved in the pathogenesis of end-stage COPD, we carried out immunohistochemical analysis to examine protein expression of NOX isoforms in the lung tissue sections of a well-characterized cohort of COPD patients undergoing lung transplantation, and in the lung tissue sections of healthy donors who were lifelong non-smokers. Intriguingly, our data demonstrate for the first time that protein expression of NOX1, NOX2, NOX4, and NOX5 was markedly increased in COPD patients compared to non-smoking donor controls. The protein expression of NOX1 was detected in bronchial epithelial cells, alveolar epithelial cells, vascular endothelial cells, and macrophages in lung tissue sections. The immunoreactivity of NOX1 was significantly upregulated in the lung sections of end-stage COPD patients compared to the donor group in all bronchial epithelial cells, alveolar epithelial cells, and vascular cells (Figure 1A,B). The protein expression of NOX2 was mostly detected in lung macrophages and neutrophils, with lower expression detected in bronchial epithelial cells and alveolar epithelial cells in both control and COPD lungs. The immunoreactivity of NOX2 was significantly higher in the lung sections of end-stage COPD patients compared to the donor controls (Figure 1C,D). The expression of NOX4 protein was also increased in bronchial epithelial cells, alveolar epithelial cells, macrophages, and vascular cells in the lung sections of end-stage COPD patients compared to the donor group (Figure 1E,F). Likewise, compared to the donor group, the expression of NOX5 in the lung sections of end-stage COPD patients was significantly upregulated in bronchial epithelial cells, alveolar epithelial cells, vascular cells, and macrophages (Figure 1G,H). These data indicate that NOX1, NOX2, NOX4, and NOX5 all remained active in the lung to contribute to oxidative stress at the end stage of severe COPD.

### 3.2. ACS Exposure Induced Upregulation of NOX Isoforms in Mouse Lungs

**Figure 2:**
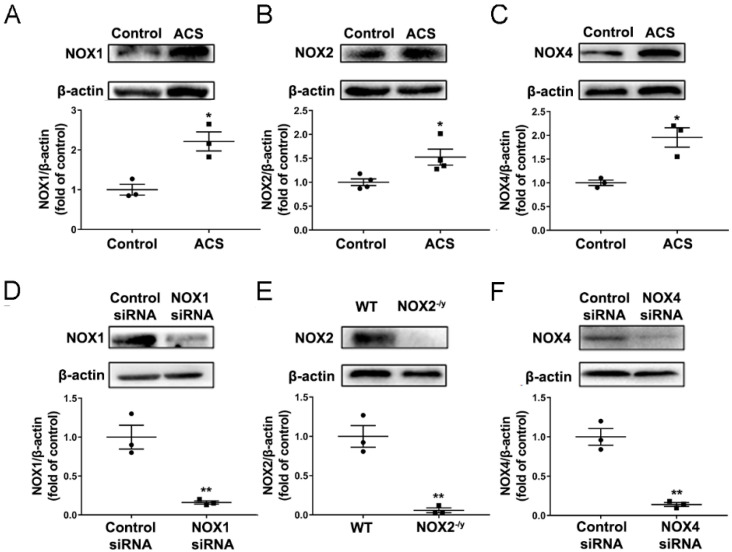
**Upregulation of NOX1, NOX2, and NOX4 in ACS exposed mouse lungs; and reduced expression of NOXs in mice genetically silenced for each isoform**. Mice were subjected to CS or filtered air for 1 h and then harvested at 6 h. The protein expression of NOX isoforms NOX1, NOX2, and NOX4 was determined in the lungs of mice exposed to ACS, mice knocked down of NOX1 or NOX4, and NOX2 knockout mice using Western blotting analyses. (**A**–**C**) Representative Western blots and grouped data of NOX1, NOX2, or NOX4 protein expression in ACS exposed mouse lungs. The data indicate that protein expression of NOX1, NOX2, and NOX4 was significantly upregulated in ACS exposed mouse lungs. (**D**–**F**) Representative Western blots and grouped data of NOX1, NOX2, or NOX4 expression in mice silenced for each isoform. The data indicate that RNAi-mediated NOX1 or NOX4 knockdown significantly attenuated NOX1 or NOX4 protein abundance respectively, while NOX2 was not detected in the lungs of NOX2^−/y^ mice. All data are presented as Mean ± SEM, *n* = 3–4. * *p* < 0.05, ** *p* < 0.01 vs. air exposed control group.

As ACS exposure has been shown to increase ROS production and inflammation in the lung [4], we sought to determine the potential effects of ACS on the expression of NOX isoforms in the mouse lung. Eight-week-old C57BL/6 male mice were exposed to CS for 1 h and then harvested at 6 h post exposure of ACS. Western blotting was used to examine protein expression of NOX1, NOX2, and NOX4 in the lung. NOX5 is not expressed in rodents and hence not examined [35]. As shown in Figure 2A–C, the protein expression of NOX1, NOX2, and NOX4 was significantly upregulated by ACS exposure in mouse lungs. To examine the specific role of each NOX isoform in mediating oxidative stress and pulmonary inflammation in response to ACS exposure, the expression of each NOX isoform in mice was knocked down using gene knockout or in vivo RNAi-mediated knockdown approaches. Indeed, RNAi-mediated NOX1 or NOX4 knockdown significantly attenuated NOX1 or NOX4 protein abundance respectively (Figure 2D,F). In addition, the NOX2 band was not detected in the lungs of NOX2^−/y^ mice (Figure 2E).

### 3.3. NOX1, NOX2, or NOX4 Deficiency Attenuated Lung ROS Production in Response to ACS Exposure

**Figure 3:**
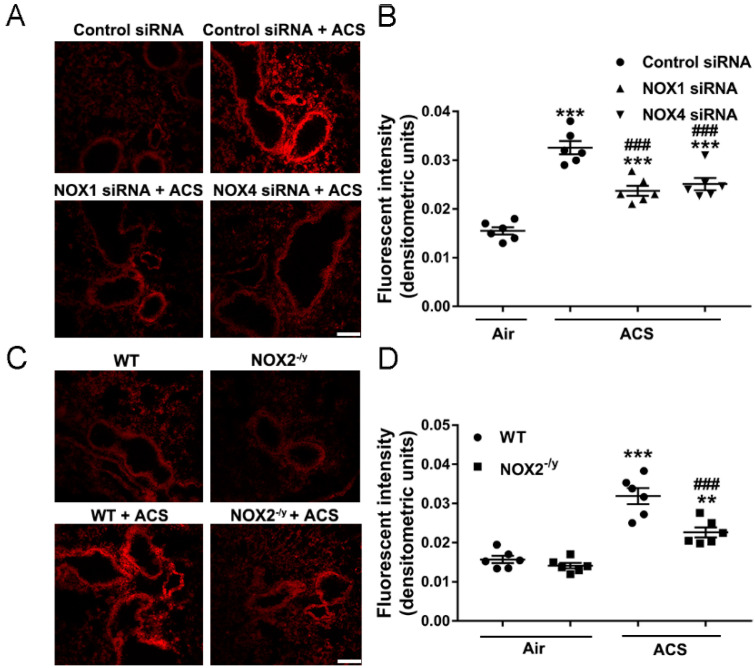
**Knockdown of NOX1, NOX2, or NOX4 significantly attenuated ACS induced ROS production in the mouse lung**. Mice were exposed to CS or filtered air for 1 h and harvested at 6 h. Frozen lung OCT sections from the superior lobe of the right lung were stained with dihydroethidium (DHE) for detection of ROS production. (**A**,**B**) Representative images and quantitative data of DHE fluorescent imaging of lung tissue sections from ACS exposed mice with or without RNAi-mediated knockdown of NOX1 or NOX4. The data indicate significantly increased ROS production in ACS exposed mice, which was markedly attenuated by RNAi-mediated in vivo silencing of NOX1 or NOX4. (**C**,**D**) Representative images and grouped data of DHE fluorescent imaging of lung tissue sections from ACS exposed wildtype (WT) mice and NOX2^−/y^ mice. The data indicate significantly increased ROS production in ACS exposed WT mice, which was markedly attenuated in NOX2^−/y^ mice. All data are presented as Mean ± SEM, *n* = 6. Scale bar, 100 µm. ** *p* < 0.01, *** *p* < 0.001 vs. air exposed control group. ^###^ *p* < 0.001 vs. ACS exposed group.

The pivotal role of oxidative stress in the development of COPD has been established. To examine the potential roles of NOX isoforms in ACS induced oxidative stress, DHE fluorescent imaging was used to detect ROS production in freshly prepared frozen lung tissue sections. Of note, ACS exposure markedly increased ROS production in the mouse lung, which was substantially attenuated in mice knocked down of NOX1 or NOX4 using in vivo RNA interference (Figure 3A,B). Likewise, ACS induced ROS production was also significantly attenuated in the mouse lung of NOX2^−/y^ mice compared to those of ACS exposed wildtype (WT) control mice (Figure 3C,D). Of note, ACS induced ROS production was not attenuated to baseline in the lung tissues of NOX1 or NOX4 siRNAtreated mice or NOX2^−/y^ mice compared to air exposed control mice. These data imply that synergistic inhibition of NOX1, NOX2, and NOX4 simultaneously might be necessary to completely alleviate ACS induced ROS production in mouse lungs.

### 3.4. NOX1 or NOX4 Silencing Decreased Inflammatory Cell Influx into the Lungs in Response to ACS Exposure

**Figure 4:**
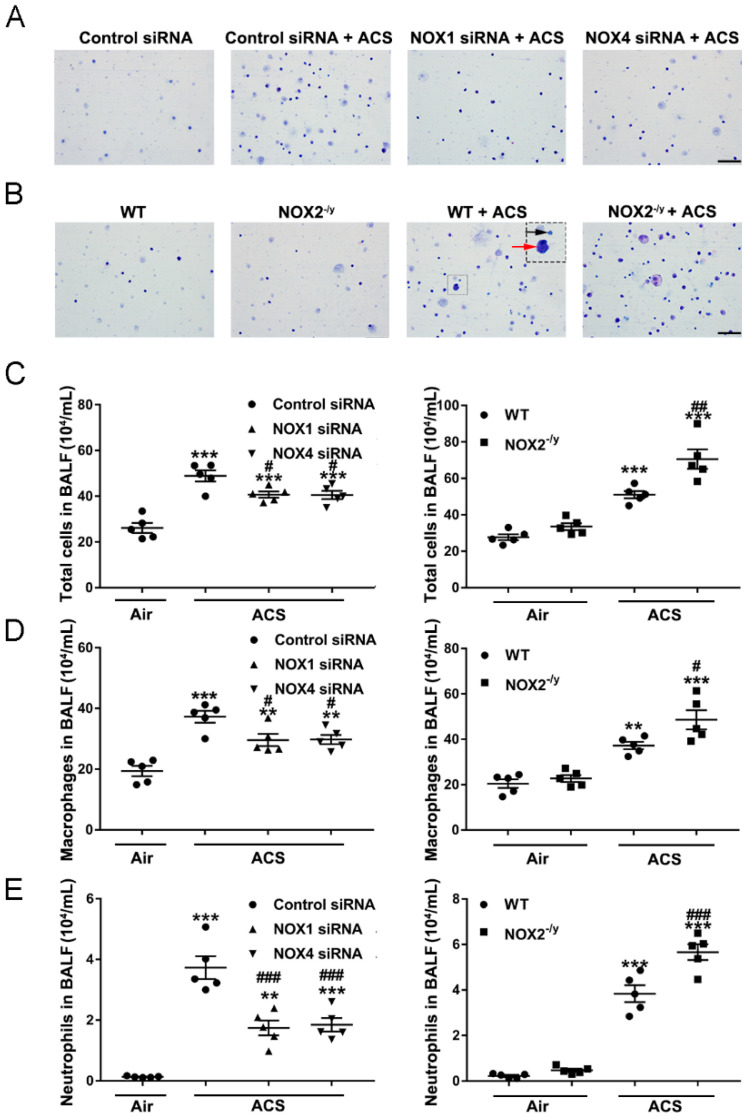
**Knockdown of NOX1 or NOX4 in mice attenuated ACS induced inflammatory cell accumulation in BALF**. Mice were exposed to CS or filtered air for 1 h, and BALF was collected at 6 h. Cells isolated from BALF were counted with a hemocytometer and stained with Giemsa. (**A**) Representative images of cells in BALF from ACS exposed mice transfected with control siRNA, NOX1 siRNA, or NOX4 siRNA. (**B**) Representative images of cells in BALF from ACS exposed WT mice and NOX2^−/y^ mice. (**C**) Total cell counts in BALF in ACS exposed mice silenced or knocked out of NOX1, NOX2, or NOX4 expression. (**D**) The number of macrophages (red arrowhead) in ACS exposed mice silenced or knocked out of NOX1, NOX2, or NOX4 expression. (**E**) The number of neutrophils (black arrowhead) in BALF in ACS exposed mice silenced or knocked out of NOX1, NOX2, or NOX4 expression. All data are presented as Mean ± SEM, *n* = 5. Scale bar, 50 µm. ** *p* < 0.01, *** *p* < 0.001 vs. air exposed control group. ^#^ *p* < 0.05, ^##^ *p* < 0.01, ^###^ *p* < 0.001 vs. ACS exposed group.

Since the lung inflammatory response is known to be downstream of oxidative stress in the mediation of COPD development [5], we investigated whether inhibition of NOX isoforms is protective against ACS induced lung inflammation in vivo. The progression of COPD is associated with accumulation and activation of inflammatory cells in bronchoalveolar lavage fluid (BALF) [36]. Previous studies have documented increased recruitment of neutrophils and macrophages to the lung upon exposure to cigarette smoke [4]. In the present study, we assessed the effects of ACS exposure on inflammatory cell accumulation in BALF collected after ACS exposure. Differential cell counts demonstrated that exposure to ACS resulted in increased numbers of macrophages and neutrophils in the BALF of WT mice, whereas it was augmented in NOX2^−/y^ mice (Figure 4A,C–E). Conversely, compared to the control siRNA-transfected mice, mice knocked down of NOX1 or NOX4 displayed a marked decrease in the number of inflammatory cells accumulated in BALF (Figure 4B–E). Similar to responses in ROS production, NOX1 or NOX4 siRNA did not attenuate inflammatory cell accumulation in BALF to baseline levels, indicating that synergistic inhibition of NOX1 and NOX4 simultaneously might be necessary to fully alleviate inflammatory cell accumulation in response to ACS exposure.

**Figure 5:**
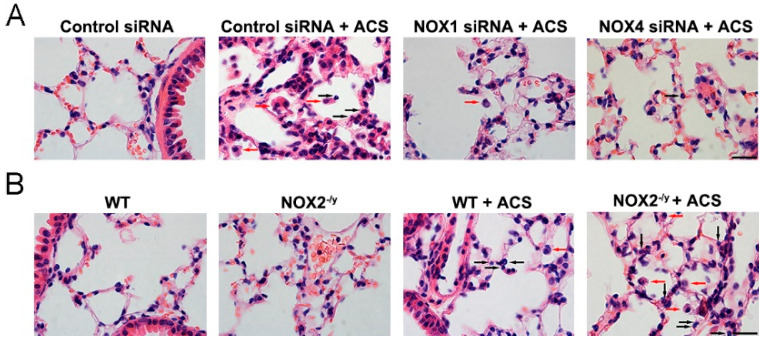
**Knockdown of NOX1 or NOX4 in mice attenuated ACS induced lung inflammation**. Mice were exposed to CS or filtered air for 1 h and harvested at 6 h. Lung tissue sections were stained with H&E, and the data indicate an increase in the numbers of macrophages (red arrowheads) and neutrophils (black arrowheads) infiltrating alveolar spaces in response to ACS in control siRNA-transfected or WT control mice. (**A**) Representative H&E images indicating that ACS exposed mice silenced of NOX1 or NOX4 expression had reduced inflammatory cell infiltration compared to control siRNA-transfected mice. (**B**) Representative H&E images indicating that ACS exposed NOX2^−/y^ mice had increased inflammatory cell infiltration compared to WT control mice. Scale bar, 20 µm.

In our study, results from H&E staining of lung tissue sections demonstrate a significant increase in infiltrating macrophages and neutrophils at alveolar spaces in response to ACS exposure (Figure 5A,B). In addition, ACS induced infiltration of inflammatory cells was markedly abrogated by NOX1 or NOX4 silencing in vivo but aggravated by NOX2 knockout (Figure 5A,B). These data suggest that NOX1 or NOX4 mediates inflammatory cell influx into the lungs in response to ACS exposure. Conversely, there was increased lung inflammation in NOX2^−/y^ mice as compared to WT mice in response to ACS exposure.

### 3.5. NOX1 or NOX4 Silencing Decreased Expression of Proinflammatory Mediators in ACS Exposed Mouse Lung

**Figure 6:**
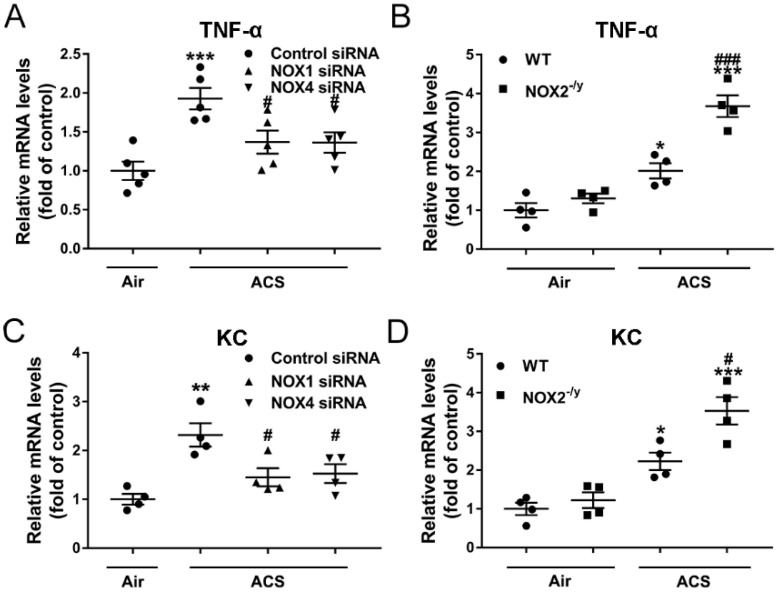
**Knockdown of NOX1 or NOX4 in mice attenuated ACS induced upregulation of pro-inflammatory mediators in the mouse lung**. Mice were exposed to CS or filtered air for 1 h and harvested at 6 h. The mRNA expression levels of (**A**,**B**) TNF-α and (**C**,**D**) KC in the lungs of ACS exposed mice silenced or knocked down of NOX1, NOX2, or NOX4 were determined by RT-qPCR. The data indicate that ACS induced upregulation of TNF-α/KC in mouse lungs was significantly attenuated in NOX1- or NOX4-silenced mice, while it was increased in NOX2^−/y^ mice. All data are presented as Mean ± SEM, *n* = 4–5. * *p* < 0.05, ** *p* < 0.01, *** *p* < 0.001 vs. air exposed control group. ^#^ *p* < 0.05, ^###^ *p* < 0.001 vs. ACS exposed group.

It has been shown that TNF-α is responsible for the majority of inflammatory cell influx and alveolar enlargement in CS-exposed mice [37]. KC, a functional homolog of human interleukin-8 in mice, is known to specifically recruit neutrophils to the airways [38]. To further evaluate the potential roles of NOX isoforms in regulating ACS induced inflammation, gene expression profiles of proinflammatory factors were determined. The mRNA abundance of TNF-α and KC was doubled in ACS exposed mice, while it was significantly attenuated in NOX1 or NOX4 siRNA-treated mice (Figure 6A,C). Similar to inflammatory cell accumulation in BALF and inflammatory cell infiltration in lung tissues described above, NOX2 knockout instead further augmented mRNA expression of TNF-α and KC (Figure 6B,D). Taken together, these data indicate that NOX1 or NOX4 silencing is effective in attenuating ACS induced induction of pro-inflammatory mediators, whereas NOX2 knockout increases the susceptibility to lung inflammation in ACS exposed mice.

### 3.6. NOX1 or NOX4 Silencing Attenuated Activation of NF-κB/COX-2 Pathway in ACS Exposed Mouse Lung

**Figure 7:**
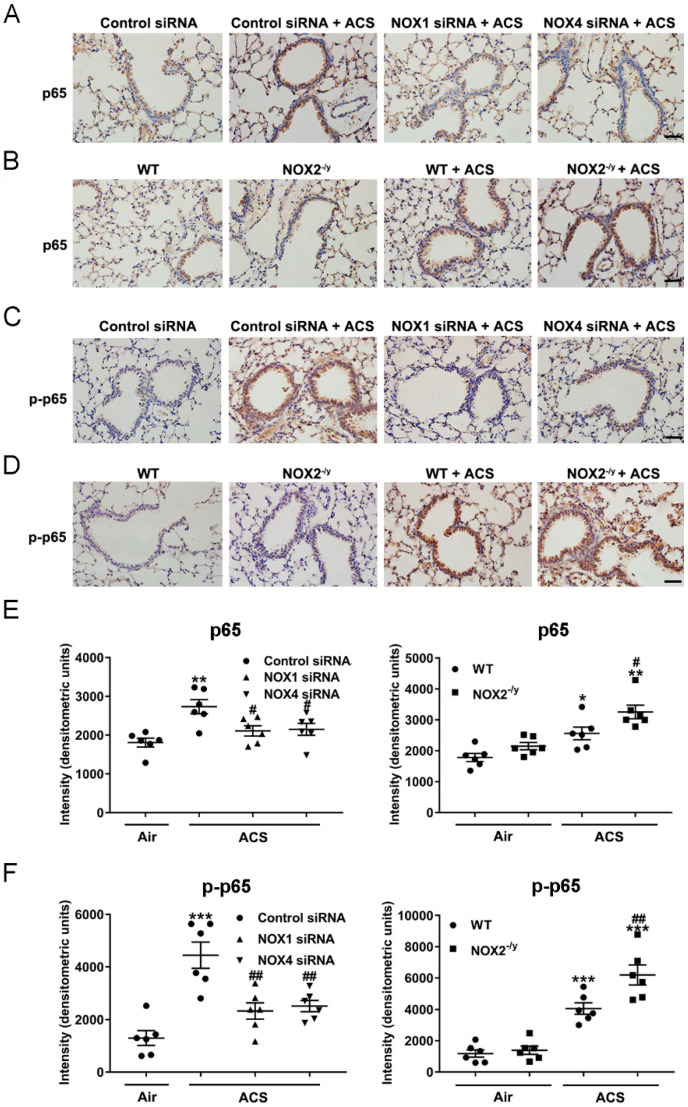
**Knockdown of NOX1 or NOX4 attenuated ACS induced NF-κB activation in the mouse lung**. Mice were exposed to CS or filtered air for 1 h and harvested at 6 h. Shown are representative immunohistochemical images of p65 (**A**,**B**) and p-p65 (**C**,**D**) expression in ACS exposed mice silenced or knocked out of NOX1, NOX2, or NOX4. Quantitative data of p65 and p-p65 expression in ACS exposed mice silenced or knocked out of NOX1, NOX2, or NOX4 are shown in panels (**E**,**F**), respectively. The data indicate that ACS induced activation of NF-κB was significantly attenuated in NOX1- or NOX4-silenced mice, while it was increased in NOX2^−/y^ mice. All data are presented as Mean ± SEM, *n* = 6. Scale bar, 50 µm. * *p* < 0.05, ** *p* < 0.01, *** *p* < 0.001 vs. air exposed control group. ^#^ *p* < 0.05, ^##^ *p* < 0.01 vs. ACS exposed group.

**Figure 8:**
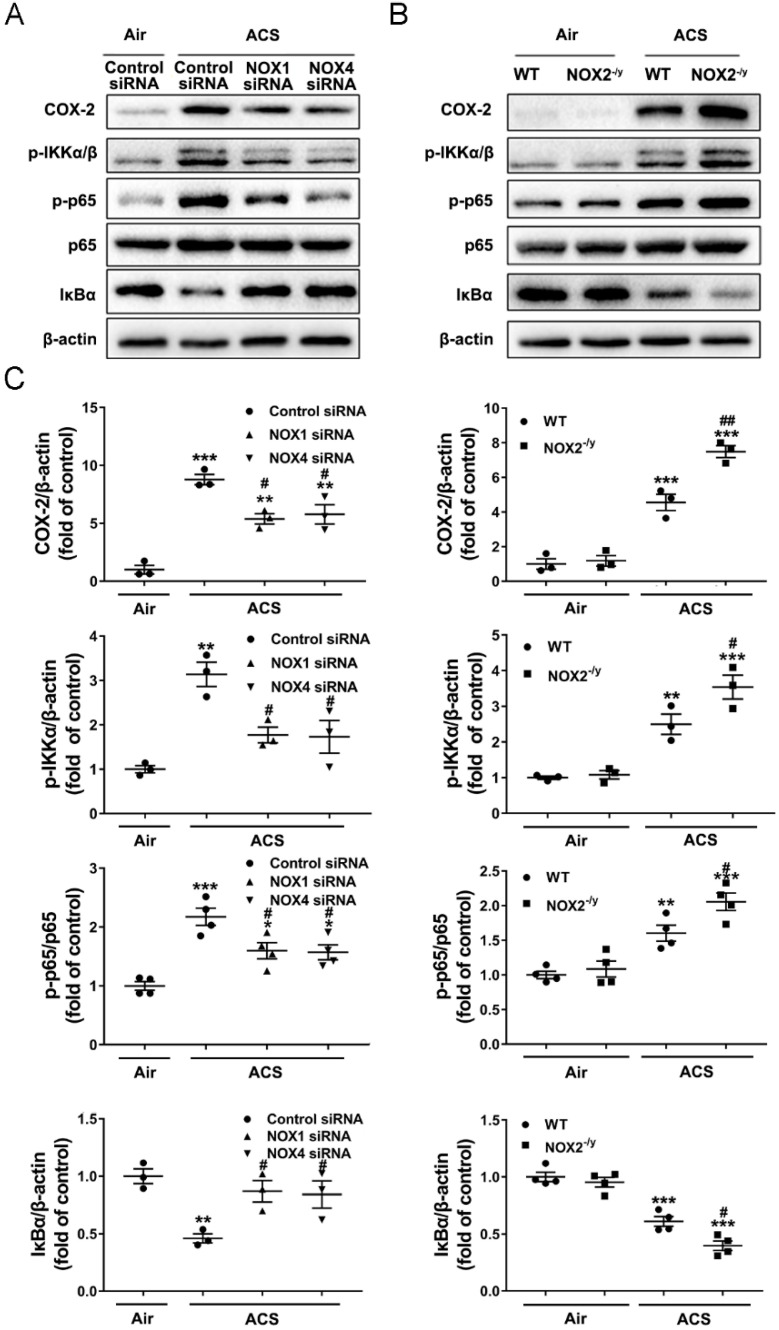
**Knockdown of NOX1 or NOX4 attenuated ACS induced activation of the NF-κB-COX-2 pathway in the mouse lung**. Mice were exposed to CS or filtered air for 1 h and harvested at 6 h. Western blotting was used to examine COX-2, p-IKKα/β, p-p65, p65, and IκBα protein expression in mouse lungs. (**A**,**B**) Representative Western blots of COX-2, p-IKKα/β, p-p65, p65, and IκBα protein expression in ACS exposed mice silenced or knocked out of NOX1, NOX2, or NOX4. (**C**) Grouped data of COX-2, p-IKKα/β, p-p65, p65, and IκBα protein expression in ACS exposed mice silenced or knocked out of NOX1, NOX2, or NOX4. The data indicate that ACS induced activation of the NF-κB-COX-2 pathway was significantly attenuated in NOX1- or NOX4-silenced mice, while it was increased in NOX2^−/y^ mice. All data are presented as Mean ± SEM, *n* = 3–5. * *p* < 0.05, ** *p* < 0.01, *** *p* < 0.001 vs. air exposed control group. ^#^ *p* < 0.05, ^##^ *p* < 0.01 vs. ACS exposed group.

Increased production of ROS has been implicated in the initiation of ACS induced lung inflammation through activation of NF-κB [39]. The NF-κB family comprises homo- and heterodimers of RelA/p65, c-Rel, RelB, NF-κB1/(p105/p50), and NF-κB2/(p100/p52) [40]. The activation of NF-κB involves phosphorylation of IκBα, which is a negative regulator of NF-κB [41]. COX-2 is a key inflammation associated enzyme regulated by NF-κB, and known to regulate lung inflammation in response to a variety of inflammatory activators [42]. We investigated potential protective effects of NOX inhibition on the NF-κB-COX-2 pathway in ACS exposed mouse lungs. The results of immunohistochemical staining indicate that ACS exposure increased levels of total p65 and phosphorylated p65 (p-p65, active form) in both bronchial epithelium and alveolar epithelial cells (Figure 7). This response was effectively alleviated in NOX1 or NOX4 siRNA-treated mice (Figure 7A,C,E,F), but further augmented in NOX2^−/y^ mice (Figure 7B,D–F). Likewise, Western blotting analyses further revealed a significant decrease in the protein abundance of IκBα, as well as a marked increase in the ratio of p-p65/p65 and p-IKKα/β and COX-2 protein levels, in the lungs of ACS exposed mice. Interestingly, these responses were all substantially attenuated in ACS exposed mice knocked down of NOX1 or NOX4 by in vivo RNA interference (Figure 8A,C). Of note, COX-2 protein abundance and the p-p65/p65 ratio were not reduced to baseline by either NOX1 or NOX4 siRNA alone, indicating that synergistic inhibition of NOX1 and NOX4 simultaneously might be necessary to fully alleviate the activation of these inflammatory responses. By contrast, the NF-κB-COX-2 pathway was further activated in ACS exposed NOX2^−/y^ mice (Figure 8B,C). Taken together, these results indicate that NOX1 and NOX4 activation mediates *early* oxidative and inflammatory responses in ACS exposed mouse lungs, while knockout of NOX2 leads to exaggeration of inflammatory responses, which is consistent with previous observations [28]. In contrast, in *end-stage* COPD patients, the NOX isoforms NOX1, NOX2, NOX4, and NOX5 all remained active to contribute to sustained oxidative stress and pathogenesis of the advanced disease.

## 4. Discussion

In the present study, we investigated the expression profiles of NOX isoforms in end-stage COPD patients and the potential differential roles of NOX isoforms in ACS induced oxidative stress and lung inflammation in mice as a model for early COPD or early-stage COPD. The most significant findings include: (1) protein expression of NOX1, NOX2, NOX4, and NOX5 is upregulated in the lung tissue sections of end-stage COPD patients, indicating that all of the NOX isoforms remain active to play important roles in the pathological development of COPD through the end stage; (2) protein expression of NOX1, NOX2, and NOX4 is upregulated in the ACS exposed mouse lung, indicating roles of NOX isoforms in acute responses to ACS that are relevant to the pathological processes of early or earlystage COPD; (3) NOX1, NOX2, or NOX4 knockdown/knockout attenuates lung ROS production in response to ACS exposure; (4) NOX1 or NOX4 knockdown protects against ACS induced lung inflammation by decreasing inflammatory cell influx and infiltration, as well as downregulating mRNA expression of TNF-α and KC, whereas NOX2 knockout enhances ACS induced inflammatory responses. These data demonstrate novel therapeutic potential of targeting NOX1 and NOX4 for the attenuation of oxidative and inflammatory responses during the early stage of COPD development, whereas for the treatment of end-stage COPD, inhibition of NOX isoforms 1, 2, 4, and 5 is necessary. Our data reveal the previously undocumented involvement of NOX enzymes in advanced/late-stage COPD. We found that NOX1, NOX4, and NOX5 were expressed in bronchial epithelial cells, alveolar epithelial cells, vascular cells, and macrophages, whereas NOX2 was mainly expressed in macrophages and neutrophils as expected, since NOX2 was initially discovered in these cells as the first characterized NOX isoform [14]. The protein expression of NOX1/2/4/5 was significantly increased by 2-fold in the lung tissue sections of patients with end-stage COPD compared to the non-smoking donor controls. These data indicate that multiple NOX isoforms remain active in the lung tissues of end-stage COPD patients, playing important roles in the pathogenesis of the advanced disease.

The present study addresses the roles of NOX isoforms in both end-stage COPD patients and ACS exposed mice. The ACS model has been recognized as a valid system to examine molecular mechanisms underlying CS induced lung disease [4]. ACS provokes oxidative stress and inflammatory responses in the lung [8]. Among ROS-producing enzymes, NOX isoforms have been implicated in the pathogenesis of cardiovascular diseases [10,11,12,13,14]. However, the potential differential roles of NOX isoforms in mediating ACS induced oxidative stress and lung inflammation, as well as in end-stage COPD patients, have never been previously investigated in parallel. Our results indicate that the protein expression levels of NOX1, NOX2, and NOX4 in mouse lungs were significantly increased after ACS exposure. The protein abundance of NOX isoforms NOX1, NOX2, NOX4, and NOX5 were all upregulated in end-stage COPD patients, indicating their roles in the late phase of the disease, while the results from ACS exposed mice support the mechanistic involvement of NOX1 and NOX4 in the early phase of COPD development using a model of ACS exposed mice. CS can directly activate inflammatory and structural cells in the lung, including macrophages, neutrophils, and epithelial cells, to release ROS [43,44]. We found that ROS production was markedly elevated in mouse lungs after ACS exposure. In addition, compared to ACS exposed control mice, the NOX1 or NOX4 siRNA-treated mice and NOX2^−/y^ mice exhibited significantly decreased ROS levels in the lung, indicating the roles of NOX1, NOX2, and NOX4 in mediating acute oxidative stress responses upon ACS exposure. This seems to share similarities with recent findings that the deletion of the NOX1 binding subunit NOXO1 was protective against the development of emphysema in mice [30]. Although we did not examine the roles of DUOX1 and DUOX2 in the present study, previous reports indicate that DUOX1 may have opposite roles, as DUOX1 was found to be downregulated in COPD [25,26,27].

CS provokes lung inflammation with an increase in the number of alveolar neutrophils and macrophages recruited from the circulation. These cells release multiple chemotactic mediators, which further sustain oxidative stress to trigger an inflammatory cascade, leading to lung damage [5,44]. In our study, there was a significant increase in total cell number and the number of macrophages and neutrophils in BALF, indicating infiltration of inflammatory cells. It was reported that TNF-α is central to ACS induced lung inflammation in mice [45], while KC is a chemotactic factor for neutrophils, whose number has been shown to increase upon exposure to CS [5,7]. Consistently, our data indicate a marked upregulation of the mRNA levels of pro-inflammatory cytokines TNF-α and KC in the lungs of ACS exposed mice. Importantly, inflammatory cell accumulation in BALF and TNF-α/KC mRNA upregulation in mouse lungs were all diminished in mice knocked down of NOX1 or NOX4 by in vivo RNA interference. NOX1 and NOX4 have been shown to be associated with inflammation related diseases in recent reports. For instance, NOX1 is a major source of vascular ROS and accelerates atherosclerosis in diabetes by inducing vascular dysfunction and inflammation [33,46]. Another study showed that a NOX1/4 inhibitor has renoprotective effects in diabetic mice, attributed to alleviation of oxidative stress, inflammation, and kidney damage [47]. In addition, inhibitors of NOX4 mitigate liver inflammation and fibrosis and increase insulin sensitivity in mice with diet-induced steatohepatitis [48]. Our data indicate that NOX1 and NOX4 function as important mediators of ROS production and lung inflammation in response to ACS exposure. NOX-derived ROS have been shown to be involved in the activation of the NF-κB pathway [39]. NF-κB is a master switch in the transcription of pro-inflammatory genes and plays a crucial role in lung inflammation during the pathogenesis of COPD [49]. COX-2 is an inflammation associated enzyme regulated by NF-κB, and is involved in lung inflammatory responses [42]. In the present study, we observed an increase in p65 phosphorylation, cytoplasmic IκBα degradation, and COX-2 protein expression in the lungs of ACS exposed mice, which was accompanied by increased mRNA expression of TNF-α and KC. TNF-α is known to regulate NF-κB and IL-8 (human homolog of KC) expression in alveolar epithelial cells [50]. Our data indicate that ACS induces NOX activation and consequent inflammatory responses, including the release of TNF-α to activate KC and the NF-κB-COX-2 pathway. Our findings therefore strongly demonstrate potential therapeutic application and efficacy of NOX1/NOX4 inhibition in diminishing these oxidative and inflammatory responses induced by ACS exposure, whereas targeting all of the NOX isoforms NOX1/2/4/5 might be necessary to treat COPD at the later stage. 

Conversely, we observed that ACS exposure induced inflammatory cell infiltration in BALF and upregulation of TNF-α/KC and NF-κB/COX-2 in the lungs were exaggerated in NOX2^−/y^ mice compared to WT control mice. The discrepancy between decreased ROS production and enhanced lung inflammatory response in NOX2^−/y^ mice is similar to a report indicating that NOX2^−/y^ and p47^phox−/−^ mice treated with lipopolysaccharide exhibited increased chemokine production and neutrophil infiltration of the lung tissue [51]. A possible explanation for these data is that genetic mutation in NOX2 or its subunits in patients causes chronic granulomatous disease (CGD), which is characterized by increased susceptibility to excessive inflammatory responses [52]. Similar to the pathogenesis of CGD, ingested particles cannot be destroyed in the phagosome, and the apoptotic mechanism is impaired in inflammatory cells in NOX2^−/y^ mice [53,54]. This may cause an increased release of chemotactic mediators from neutrophils and macrophages, leading to the increased influx of inflammatory cells. The increase in the NF-κB inflammatory pathway in NOX2^−/y^ mice may be in part due to the increased expression of TNF-α, an important stimulus of NF-κB. This feedback regulation may prolong and amplify inflammatory responses [55]. Importantly, our findings are consistent with Yao et al., who indicated the attenuation of ROS production and exaggerated inflammation and emphysema in NOX2 knockout animals, as discussed earlier [28], although the study by Trocme et al., on the other hand, contradicts these findings showing that NOX2 knockout animals were protected from elastase-induced emphysema [29]. Therefore, there seem to be differential roles of NOX1/4 versus NOX2 in modulating lung inflammation in response to ACS, indicating that targeted therapies against NOX1/4 specifically would be more beneficial in treating earlystage COPD.

## 5. Conclusions

Our data demonstrate for the first time that NOX isoforms NOX1, NOX2, NOX4, and NOX5 all remained active in lung tissues of end-stage COPD patients, indicating critical roles of these enzymes in the pathogenesis of the advanced disease. We also observed differential involvement of NOX1/NOX4 versus NOX2, in acute oxidative stress and inflammatory responses induced by ACS exposure, implicating selective roles of NOX1/4 activation in the early stage of COPD development. These data innovatively demonstrate that differential targeting of NOX isoforms at different stages of disease development is important for effective management of the devastating disease of COPD.

## Figures and Tables

**Table 1:** Clinical characteristics of donor controls and COPD recipients.

	Donors	COPD
Number	7	18
Males/females	5/2	17/1
Age (yr)	40 ± 4.2	60 ± 1.8 ***
Height (m)	1.66 ± 0.03	1.68 ± 0.01
Weight (kg)	65 ± 4.5	55 ± 2.3 *
Body mass index	23 ± 1.1	19 ± 0.7 *
Smoking, pack-years	0	42 ± 6.1
FEV1%, predicted	-	24 ± 4.1
FEV1/FVC, %	-	37 ± 2.6
Single/bilateral lung transplantation	-	4/14

Data are presented as Mean ± SEM unless otherwise indicated. * *p <* 0.05, *** *p <* 0.001 compared to donor controls.

## Data Availability

All of the original data for the article are available upon reasonable request.
